# Substitutional disorder in the ionic diorganoanti­mony halide adduct [bromido/chlorido(0.33/0.67)][2-(di­methyl­amino­meth­yl)phen­yl][2-(di­methyl­ammonio­meth­yl)phen­yl]anti­mony(III) 0.75-bromide 0.25-chloride

**DOI:** 10.1107/S1600536810009797

**Published:** 2010-03-20

**Authors:** Albert P. Soran, Vilma R. Bojan

**Affiliations:** a Faculty of Chemistry and Chemical Engineering, Babes-Bolyai University, Arany Janos Street, No. 11, RO-400028, Cluj Napoca, Romania

## Abstract

The title complex, [SbBr_0.33_Cl_0.67_(C_9_H_13_N)(C_9_H_12_N)]Br_0.75_Cl_0.25_, exhibits substitutional disorder of both halogen atoms in the asymmetric unit, however, with different occupancies. Thus, the halogen atom bonded to Sb has 0.67 (4) occupancy for Cl and 0.33 (4) for Br, while the anionic halogen atom shows 0.75 (4) occupancy for Br and 0.25 (4) for Cl. An N—H⋯Cl/Br hydrogen bond is established between the cation and the halide anion. The coordination geometry of the Sb center in the cation is distorted pseudo-trigonal-bipyramidal as a result of the strong intra­molecular N→Sb coordination *trans* to the Sb—Cl/Br bond. The pendant arm on the second ligand is twisted away from the metal center. The compound crystallizes as a racemate, *i.e.* a mixture of (*R*_N2_,*C*_Sb1_) and (*S*_N2_,*A*_Sb1_) isomers with respect to planar chirality induced by the coordinating N atom and chelate-induced Sb chirality. These isomers are associated through C_phen­yl_—H⋯Cl/Br hydrogen bonds, forming a three-dimensional architecture.

## Related literature

For an isostructural compound, see: Opris *et al.* (2003 ▶). For related ionic organoantimony adducts, see: Sharma *et al.* (2004 ▶). For the chirality induced by the coordination of the N atom, see: IUPAC (1979 ▶, 2005 ▶). For Sb—N distances, see: Emsley (1994 ▶).
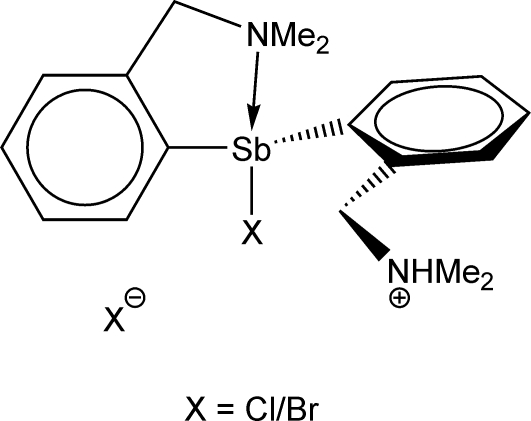


## Experimental

### 

#### Crystal data

[SbBr_0.33_Cl_0.67_(C_9_H_13_N)(C_9_H_12_N)]Br_0.75_Cl_0.25_
*M* *_r_* = 510.07
Monoclinic, 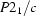

*a* = 13.8159 (19) Å
*b* = 12.6775 (18) Å
*c* = 12.5984 (17) Å
β = 105.342 (3)°
*V* = 2128.0 (5) Å^3^
*Z* = 4
Mo *K*α radiation
μ = 3.30 mm^−1^
*T* = 297 K
0.28 × 0.22 × 0.18 mm

#### Data collection

Bruker SMART APEX CCD area-detector diffractometer
Absorption correction: multi-scan (*SADABS*; Bruker, 2000 ▶) *T*_min_ = 0.458, *T*_max_ = 0.588
15150 measured reflections
3745 independent reflections
3298 reflections with *I* > 2σ(*I*)
*R*_int_ = 0.044

#### Refinement

*R*[*F* ^2^ > 2σ(*F* ^2^)] = 0.045
*wR*(*F* ^2^) = 0.112
*S* = 1.15
3745 reflections
216 parameters
2 restraints
H atoms treated by a mixture of independent and constrained refinement
Δρ_max_ = 1.47 e Å^−3^
Δρ_min_ = −0.56 e Å^−3^

### 

Data collection: *SMART* (Bruker, 2000 ▶); cell refinement: *SAINT-Plus* (Bruker, 2001 ▶); data reduction: *SAINT-Plus*; program(s) used to solve structure: *SHELXS97* (Sheldrick, 2008 ▶); program(s) used to refine structure: *SHELXL97* (Sheldrick, 2008 ▶); molecular graphics: *DIAMOND* (Brandenburg, 2006 ▶); software used to prepare material for publication: *enCIFer* (Allen *et al.*, 2004 ▶) and *publCIF* (Westrip, 2010 ▶).

## Supplementary Material

Crystal structure: contains datablocks I, global. DOI: 10.1107/S1600536810009797/zb2001sup1.cif

Structure factors: contains datablocks I. DOI: 10.1107/S1600536810009797/zb2001Isup2.hkl

Additional supplementary materials:  crystallographic information; 3D view; checkCIF report

## Figures and Tables

**Table 1:** Hydrogen-bond geometry (Å, °) *X* = Cl/Br.

*D*—H⋯*A*	*D*—H	H⋯*A*	*D*⋯*A*	*D*—H⋯*A*
N1—H1⋯*X*2^i^	0.86 (6)	2.39	3.220 (7)	164
C12—H12⋯*X*1^ii^	0.93	2.91	3.827 (6)	167
C14—H14⋯*X*1^iii^	0.93	2.86	3.766 (8)	164

Symmetry codes: (i) 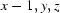; (ii) 
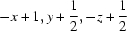
; (iii) 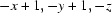.

**Fig. 1.**
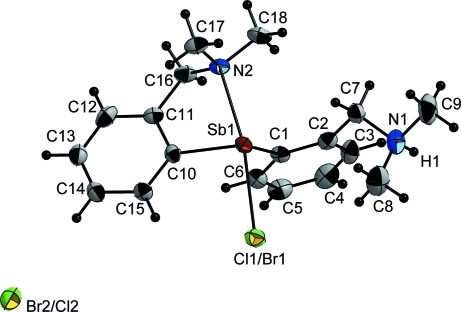
A view of the asymmetric unit showing the atom-numbering scheme at 30% probability thermal ellipsoids for the (RN2,CSb1) isomer.

**Fig. 2.**
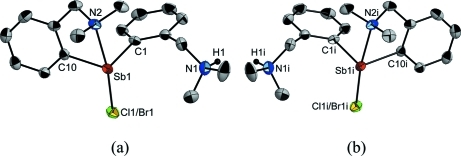
Molecular structure at 30% probability ellipsoids of (RN2,CSb1) (a) and (SN2,ASb1) (b) isomers present in crystals of the title compound. Only the cationic fragment is shown. All hydrogen atoms except H1 atoms have been omitted. Symmetry code: (i) 1-x, 1-y, -z.

**Fig. 3.**
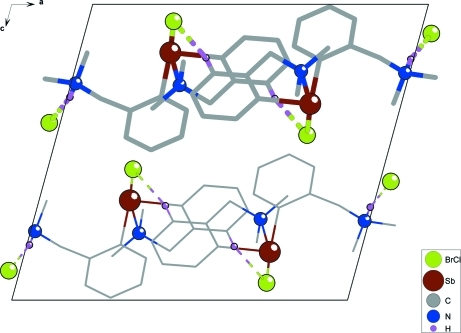
Unit cell showingpolymeric all-(RN2,CSb1) (thick lines) and all-(SN2,ASb1) (thin lines) strands formed as a result of H12···.Cl1/Br1 hydrogen bonding. All hydrogen atoms except H12 and H1 atoms have been omitted.

**Fig. 4.**
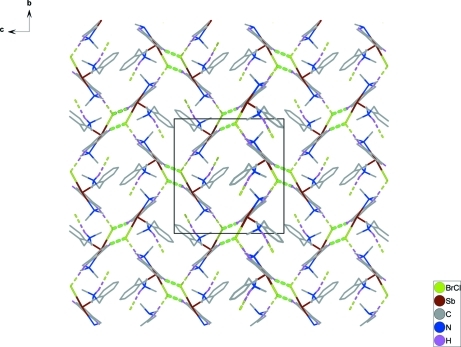
Three-dimensional networkformed bypolymeric all-(RN2,CSb1) and all-(SN2,ASb1) strands bridged by H14···Cl1/Br1 hydrogen bonds (thick green lines). All hydrogen atoms except H12, H14 and H1 atoms have been omitted.

## supplementary crystallographic information

### Comment

The chlorido/bromido[2-(dimethylaminomethyl)phenyl][2-(dimethylammoniomethyl)-phenyl]antimony chloride/bromide, [C_72_H_100_Br_4.32_Cl_3.68_N_8_Sb_4_], exhibits substitutional disorder of both halogen atoms of the asymmetric unit, however with different occupancies. Thus, the halogen bonded to Sb has 0.67 (4) occupancy for Cl and 0.33 (4) for Br while the anionic halogen shows 0.75 (4) occupancy for Br and 0.25 (4) for Cl.

A hydrogen bond is established between the cation and the halide anion [Cl2/Br2···H1 = 2.39 Å; N1—H1···Cl2/Br2 = 163.6°].

The title compound is isostructural with [{2-(Me_2_NCH_2_)C_6_H_4_}Sb{C_6_H_4_(CH_2_NHMe_2_)-2}]^+^[I]^-^ (Opris *et al*., 2003), having only a slightly smaller cell volume.

The coordination geometry of the Sb center in the cationic fragment is distorted, *peudo*-trigonal bipyramidal as a result of the strong intramolecular N→Sb coordination [Sb1—N2 = 2.414 (5) Å] *trans* to the Sb1—Cl1/Br1 bond [(N2—Sb1—Cl1/Br1 = 166.8 (1)°]. The pendant arm on the second ligand is twisted away from the metal center [non-bonding Sb1–N1 = 4.312 (6) Å] (Emsley, 1994), its N1 atom being protonated (Fig. 1.)

Coordination of N atom induces planar chirality, with the phenyl ring as chiral plane and the nitrogen as pilot atom (IUPAC, 1979). This intramolecular coordination of the nitrogen atom to antimony induces chirality at the Sb centre (IUPAC, 2005). Thus the compound crystallizes as a racemate, *i.e. *a mixture of (*R*_N2_,*C*_Sb1_) and (*S*_N2_,*A*_Sb1_) isomers (Fig. 2.), with two of each isomers in the unit cell.

Same kind of isomers form ribbon-like *all*-(*R*_N2_,*C*_Sb1_) and *all*-(*S*_N2_,*A*_Sb1_) polymers through [H12···.Cl1/Br1 = 2.91 Å] hydrogen bonds (Fig. 3.). These ribbon-like polymers are further associated throughhydrogen bonds [H14···.Cl1/Br1 = 2.86 Å] to form a three-dimensional architecture (Fig. 4.)

### Experimental

In the attempted synthesis of R_2_SbMes from mesitylmagnesium bromide and R_2_SbCl.(R = 2-Me_2_NCH_2_C_6_H_4_), crystals of the title compound were isolated from a chloroform-hexane mixture, due to partial hydrolysis followed by the protonation of one of the organic ligands.

### Refinement

All hydrogen atoms, except H1 attached to N1, were placed in calculated positions using a riding model, with C—H = 0.93–0.97 Å and with *U*iso=1.5*U*eq (C) for methyl H and *U*iso= 1.2*U*eq (C) for aryl H. The methyl groups were allowed to rotate while retaining tetrahedral geometry. The H1 hydrogen atom attached to N1 nitrogen atom was located from the difference map and the N1—H1 distance was restrained to 0.86 Å. The two halide atoms were refined as substitutional disorder between chlorine and bromine, with 0.67 occupancy for Cl and 0.33 for Br for Cl1/Br1 and 0.75 occupancy for Br and 0.25 for Cl for Cl2/Br2.

### Crystal data

**Table:**

[SbBr_0.33_Cl_0.67_(C_9_H_13_N)(C_9_H_12_N)]Br_0.75_Cl_0.25_	*F*(000) = 1000
*M**_r_* = 510.07	*D*_x_ = 1.581 Mg m^−^^3^
Monoclinic, *P*2_1_/*c*	Mo *K*α radiation, λ = 0.71073 Å
Hall symbol: -P 2ybc	Cell parameters from 3355 reflections
*a* = 13.8159 (19) Å	θ = 2.3–22.7°
*b* = 12.6775 (18) Å	µ = 3.30 mm^−^^1^
*c* = 12.5984 (17) Å	*T* = 297 K
β = 105.342 (3)°	Block, colourless
*V* = 2128.0 (5) Å^3^	0.28 × 0.22 × 0.18 mm
*Z* = 4	

### Data collection

**Table:**

Bruker SMART APEX CCD area-detector diffractometer	3745 independent reflections
Radiation source: fine-focus sealed tube	3298 reflections with *I* > 2σ(*I*)
graphite	*R*_int_ = 0.044
phi and ω scans	θ_max_ = 25.0°, θ_min_ = 2.2°
Absorption correction: multi-scan (*SADABS*; Bruker, 2000)	*h* = −16→16
*T*_min_ = 0.458, *T*_max_ = 0.588	*k* = −15→15
15150 measured reflections	*l* = −14→14

### Refinement

**Table:**

Refinement on *F*^2^	Primary atom site location: structure-invariant direct methods
Least-squares matrix: full	Secondary atom site location: difference Fourier map
*R*[*F*^2^ > 2σ(*F*^2^)] = 0.045	Hydrogen site location: inferred from neighbouring sites
*wR*(*F*^2^) = 0.112	H atoms treated by a mixture of independent and constrained refinement
*S* = 1.15	*w* = 1/[σ^2^(*F*_o_^2^) + (0.0441*P*)^2^ + 3.758*P*] where *P* = (*F*_o_^2^ + 2*F*_c_^2^)/3
3745 reflections	(Δ/σ)_max_ < 0.001
216 parameters	Δρ_max_ = 1.47 e Å^−^^3^
2 restraints	Δρ_min_ = −0.56 e Å^−^^3^

### Special details

**Table:**

Geometry. All esds (except the esd in the dihedral angle between two l.s. planes) are estimated using the full covariance matrix. The cell esds are taken into account individually in the estimation of esds in distances, angles and torsion angles; correlations between esds in cell parameters are only used when they are defined by crystal symmetry. An approximate (isotropic) treatment of cell esds is used for estimating esds involving l.s. planes.
Refinement. Refinement of *F*^2^ against ALL reflections. The weighted *R*-factor wR and goodness of fit *S* are based on *F*^2^, conventional *R*-factors *R* are based on *F*, with *F* set to zero for negative *F*^2^. The threshold expression of *F*^2^ > σ(*F*^2^) is used only for calculating *R*-factors(gt) etc. and is not relevant to the choice of reflections for refinement. *R*-factors based on *F*^2^ are statistically about twice as large as those based on *F*, and *R*- factors based on ALL data will be even larger.

### Fractional atomic coordinates and isotropic or equivalent isotropic displacement parameters (Å^2^)

**Table:**

	*x*	*y*	*z*	*U*_iso_*/*U*_eq_	Occ. (<1)
Br2	0.96752 (7)	0.33618 (8)	0.40075 (9)	0.0779 (3)	0.75
Br1	0.25490 (8)	0.45952 (8)	0.05792 (9)	0.0561 (3)	0.33
C1	0.2796 (4)	0.5553 (4)	0.3124 (4)	0.0415 (13)	
C2	0.1990 (5)	0.5470 (5)	0.3592 (5)	0.0474 (14)	
C3	0.2080 (5)	0.4808 (5)	0.4502 (5)	0.0593 (17)	
H3	0.1550	0.4756	0.4825	0.071*	
C4	0.2929 (6)	0.4238 (6)	0.4923 (5)	0.069 (2)	
H4	0.2968	0.3790	0.5517	0.082*	
C5	0.3730 (5)	0.4323 (5)	0.4473 (5)	0.0623 (18)	
H5	0.4315	0.3943	0.4770	0.075*	
C6	0.3662 (5)	0.4972 (5)	0.3581 (5)	0.0503 (15)	
H6	0.4204	0.5024	0.3277	0.060*	
C7	0.1020 (5)	0.6079 (5)	0.3195 (5)	0.0540 (16)	
H7A	0.0884	0.6451	0.3813	0.065*	
H7B	0.1100	0.6601	0.2662	0.065*	
C8	0.0235 (6)	0.4925 (8)	0.1645 (6)	0.094 (3)	
H8A	−0.0344	0.4499	0.1326	0.141*	
H8B	0.0827	0.4494	0.1790	0.141*	
H8C	0.0285	0.5478	0.1142	0.141*	
C9	−0.0813 (6)	0.6012 (8)	0.2520 (9)	0.100 (3)	
H9A	−0.0800	0.6602	0.2046	0.150*	
H9B	−0.0867	0.6264	0.3221	0.150*	
H9C	−0.1379	0.5572	0.2195	0.150*	
C10	0.4296 (4)	0.6542 (4)	0.1811 (5)	0.0414 (13)	
C11	0.4834 (4)	0.7263 (4)	0.2586 (5)	0.0435 (13)	
C12	0.5832 (5)	0.7454 (5)	0.2665 (6)	0.0598 (17)	
H12	0.6190	0.7925	0.3190	0.072*	
C13	0.6304 (5)	0.6950 (5)	0.1970 (6)	0.0594 (17)	
H13	0.6975	0.7094	0.2022	0.071*	
C14	0.5804 (5)	0.6252 (5)	0.1218 (6)	0.0564 (16)	
H14	0.6131	0.5908	0.0759	0.068*	
C15	0.4796 (4)	0.6046 (5)	0.1128 (5)	0.0481 (14)	
H15	0.4451	0.5569	0.0602	0.058*	
C16	0.4306 (5)	0.7800 (5)	0.3341 (5)	0.0559 (16)	
H16A	0.4337	0.7359	0.3979	0.067*	
H16B	0.4634	0.8465	0.3595	0.067*	
C17	0.3179 (6)	0.8851 (5)	0.1948 (6)	0.0618 (18)	
H17A	0.3399	0.9496	0.2336	0.093*	
H17B	0.2495	0.8926	0.1521	0.093*	
H17C	0.3596	0.8696	0.1468	0.093*	
C18	0.2643 (6)	0.8243 (6)	0.3521 (6)	0.0659 (19)	
H18A	0.2700	0.7678	0.4042	0.099*	
H18B	0.1952	0.8329	0.3122	0.099*	
H18C	0.2885	0.8884	0.3905	0.099*	
Cl1	0.25490 (8)	0.45952 (8)	0.05792 (9)	0.0561 (3)	0.67
Cl2	0.96752 (7)	0.33618 (8)	0.40075 (9)	0.0779 (3)	0.25
H1	0.008 (6)	0.492 (5)	0.315 (5)	0.08 (3)*	
N1	0.0135 (4)	0.5386 (5)	0.2675 (5)	0.0636 (15)	
N2	0.3249 (4)	0.7990 (4)	0.2740 (4)	0.0486 (12)	
Sb1	0.27062 (3)	0.64376 (3)	0.16318 (3)	0.03907 (15)	

### Atomic displacement parameters (Å^2^)

**Table:**

	*U*^11^	*U*^22^	*U*^33^	*U*^12^	*U*^13^	*U*^23^
Br2	0.0744 (6)	0.0738 (6)	0.0876 (7)	−0.0200 (5)	0.0252 (5)	−0.0053 (5)
Br1	0.0655 (7)	0.0544 (6)	0.0513 (6)	0.0023 (5)	0.0203 (5)	−0.0077 (5)
C1	0.053 (3)	0.041 (3)	0.030 (3)	0.002 (3)	0.010 (2)	0.000 (2)
C2	0.052 (4)	0.049 (3)	0.039 (3)	−0.006 (3)	0.009 (3)	−0.002 (3)
C3	0.064 (4)	0.073 (5)	0.043 (3)	−0.011 (4)	0.017 (3)	0.009 (3)
C4	0.085 (5)	0.071 (5)	0.042 (4)	−0.007 (4)	0.004 (4)	0.015 (3)
C5	0.069 (5)	0.053 (4)	0.054 (4)	0.007 (3)	−0.004 (3)	0.011 (3)
C6	0.050 (4)	0.054 (4)	0.046 (3)	0.004 (3)	0.012 (3)	0.001 (3)
C7	0.056 (4)	0.057 (4)	0.056 (4)	0.000 (3)	0.026 (3)	0.003 (3)
C8	0.077 (6)	0.135 (8)	0.064 (5)	−0.026 (5)	0.010 (4)	−0.011 (5)
C9	0.044 (4)	0.111 (7)	0.145 (9)	0.006 (4)	0.023 (5)	0.025 (6)
C10	0.042 (3)	0.044 (3)	0.040 (3)	0.002 (2)	0.013 (2)	0.004 (2)
C11	0.050 (3)	0.039 (3)	0.039 (3)	0.004 (3)	0.006 (3)	0.003 (2)
C12	0.054 (4)	0.052 (4)	0.063 (4)	−0.010 (3)	−0.002 (3)	0.005 (3)
C13	0.048 (4)	0.056 (4)	0.075 (5)	−0.001 (3)	0.018 (3)	0.013 (3)
C14	0.052 (4)	0.061 (4)	0.062 (4)	0.008 (3)	0.024 (3)	0.005 (3)
C15	0.050 (4)	0.052 (3)	0.041 (3)	0.000 (3)	0.012 (3)	−0.001 (3)
C16	0.062 (4)	0.052 (4)	0.049 (4)	0.000 (3)	0.007 (3)	−0.013 (3)
C17	0.076 (5)	0.040 (3)	0.067 (4)	0.003 (3)	0.015 (4)	0.003 (3)
C18	0.082 (5)	0.061 (4)	0.059 (4)	0.008 (4)	0.026 (4)	−0.018 (3)
Cl1	0.0655 (7)	0.0544 (6)	0.0513 (6)	0.0023 (5)	0.0203 (5)	−0.0077 (5)
Cl2	0.0744 (6)	0.0738 (6)	0.0876 (7)	−0.0200 (5)	0.0252 (5)	−0.0053 (5)
N1	0.050 (3)	0.076 (4)	0.067 (4)	−0.001 (3)	0.018 (3)	0.013 (3)
N2	0.062 (3)	0.041 (3)	0.044 (3)	0.004 (2)	0.016 (2)	−0.006 (2)
Sb1	0.0428 (2)	0.0423 (2)	0.0326 (2)	0.00455 (16)	0.01080 (16)	0.00087 (16)

### Geometric parameters (Å, °)

**Table:**

Br1—Sb1	2.6662 (11)	C10—C11	1.398 (8)
C1—C6	1.393 (8)	C10—Sb1	2.152 (6)
C1—C2	1.396 (8)	C11—C12	1.378 (9)
C1—Sb1	2.164 (5)	C11—C16	1.507 (8)
C2—C3	1.400 (8)	C12—C13	1.379 (9)
C2—C7	1.512 (9)	C12—H12	0.9300
C3—C4	1.359 (10)	C13—C14	1.347 (9)
C3—H3	0.9300	C13—H13	0.9300
C4—C5	1.375 (10)	C14—C15	1.392 (9)
C4—H4	0.9300	C14—H14	0.9300
C5—C6	1.376 (8)	C15—H15	0.9300
C5—H5	0.9300	C16—N2	1.476 (8)
C6—H6	0.9300	C16—H16A	0.9700
C7—N1	1.507 (8)	C16—H16B	0.9700
C7—H7A	0.9700	C17—N2	1.465 (8)
C7—H7B	0.9700	C17—H17A	0.9600
C8—N1	1.463 (10)	C17—H17B	0.9600
C8—H8A	0.9600	C17—H17C	0.9600
C8—H8B	0.9600	C18—N2	1.486 (8)
C8—H8C	0.9600	C18—H18A	0.9600
C9—N1	1.500 (9)	C18—H18B	0.9600
C9—H9A	0.9600	C18—H18C	0.9600
C9—H9B	0.9600	N1—H1	0.86 (6)
C9—H9C	0.9600	N2—Sb1	2.414 (5)
C10—C15	1.388 (8)		
			
C6—C1—C2	118.6 (5)	C14—C13—C12	120.7 (6)
C6—C1—Sb1	118.6 (4)	C14—C13—H13	119.6
C2—C1—Sb1	122.5 (4)	C12—C13—H13	119.6
C1—C2—C3	118.9 (6)	C13—C14—C15	119.7 (6)
C1—C2—C7	123.9 (5)	C13—C14—H14	120.1
C3—C2—C7	117.2 (6)	C15—C14—H14	120.1
C4—C3—C2	121.3 (6)	C10—C15—C14	121.1 (6)
C4—C3—H3	119.4	C10—C15—H15	119.5
C2—C3—H3	119.4	C14—C15—H15	119.5
C3—C4—C5	120.2 (6)	N2—C16—C11	109.2 (5)
C3—C4—H4	119.9	N2—C16—H16A	109.8
C5—C4—H4	119.9	C11—C16—H16A	109.8
C4—C5—C6	119.6 (6)	N2—C16—H16B	109.8
C4—C5—H5	120.2	C11—C16—H16B	109.8
C6—C5—H5	120.2	H16A—C16—H16B	108.3
C5—C6—C1	121.4 (6)	N2—C17—H17A	109.5
C5—C6—H6	119.3	N2—C17—H17B	109.5
C1—C6—H6	119.3	H17A—C17—H17B	109.5
N1—C7—C2	113.1 (5)	N2—C17—H17C	109.5
N1—C7—H7A	109.0	H17A—C17—H17C	109.5
C2—C7—H7A	109.0	H17B—C17—H17C	109.5
N1—C7—H7B	109.0	N2—C18—H18A	109.5
C2—C7—H7B	109.0	N2—C18—H18B	109.5
H7A—C7—H7B	107.8	H18A—C18—H18B	109.5
N1—C8—H8A	109.5	N2—C18—H18C	109.5
N1—C8—H8B	109.5	H18A—C18—H18C	109.5
H8A—C8—H8B	109.5	H18B—C18—H18C	109.5
N1—C8—H8C	109.5	C8—N1—C9	112.3 (7)
H8A—C8—H8C	109.5	C8—N1—C7	111.3 (5)
H8B—C8—H8C	109.5	C9—N1—C7	109.2 (6)
N1—C9—H9A	109.5	C8—N1—H1	113 (5)
N1—C9—H9B	109.5	C9—N1—H1	103 (5)
H9A—C9—H9B	109.5	C7—N1—H1	108 (5)
N1—C9—H9C	109.5	C17—N2—C16	110.4 (5)
H9A—C9—H9C	109.5	C17—N2—C18	110.0 (5)
H9B—C9—H9C	109.5	C16—N2—C18	110.5 (5)
C15—C10—C11	118.0 (5)	C17—N2—Sb1	105.0 (4)
C15—C10—Sb1	124.6 (4)	C16—N2—Sb1	106.1 (3)
C11—C10—Sb1	116.9 (4)	C18—N2—Sb1	114.6 (4)
C12—C11—C10	120.2 (6)	C10—Sb1—C1	96.8 (2)
C12—C11—C16	121.2 (6)	C10—Sb1—N2	74.76 (19)
C10—C11—C16	118.6 (5)	C1—Sb1—N2	88.97 (18)
C11—C12—C13	120.3 (6)	C10—Sb1—Br1	93.01 (15)
C11—C12—H12	119.8	C1—Sb1—Br1	87.51 (14)
C13—C12—H12	119.8	N2—Sb1—Br1	166.78 (12)
			
C6—C1—C2—C3	0.0 (8)	C2—C7—N1—C8	−66.9 (7)
Sb1—C1—C2—C3	174.4 (4)	C2—C7—N1—C9	168.5 (6)
C6—C1—C2—C7	178.3 (6)	C11—C16—N2—C17	−73.5 (6)
Sb1—C1—C2—C7	−7.3 (8)	C11—C16—N2—C18	164.6 (5)
C1—C2—C3—C4	−0.9 (10)	C11—C16—N2—Sb1	39.8 (5)
C7—C2—C3—C4	−179.3 (6)	C15—C10—Sb1—C1	112.6 (5)
C2—C3—C4—C5	1.5 (11)	C11—C10—Sb1—C1	−75.7 (4)
C3—C4—C5—C6	−1.2 (11)	C15—C10—Sb1—N2	−160.4 (5)
C4—C5—C6—C1	0.3 (10)	C11—C10—Sb1—N2	11.3 (4)
C2—C1—C6—C5	0.3 (9)	C15—C10—Sb1—Br1	24.7 (5)
Sb1—C1—C6—C5	−174.3 (5)	C11—C10—Sb1—Br1	−163.6 (4)
C1—C2—C7—N1	112.2 (6)	C6—C1—Sb1—C10	−22.0 (5)
C3—C2—C7—N1	−69.5 (7)	C2—C1—Sb1—C10	163.7 (5)
C15—C10—C11—C12	−0.9 (8)	C6—C1—Sb1—N2	−96.5 (5)
Sb1—C10—C11—C12	−173.1 (4)	C2—C1—Sb1—N2	89.1 (5)
C15—C10—C11—C16	−179.4 (5)	C6—C1—Sb1—Br1	70.8 (4)
Sb1—C10—C11—C16	8.3 (7)	C2—C1—Sb1—Br1	−103.6 (4)
C10—C11—C12—C13	1.1 (9)	C17—N2—Sb1—C10	88.5 (4)
C16—C11—C12—C13	179.6 (6)	C16—N2—Sb1—C10	−28.5 (4)
C11—C12—C13—C14	−1.1 (10)	C18—N2—Sb1—C10	−150.6 (5)
C12—C13—C14—C15	0.9 (10)	C17—N2—Sb1—C1	−174.2 (4)
C11—C10—C15—C14	0.6 (9)	C16—N2—Sb1—C1	68.8 (4)
Sb1—C10—C15—C14	172.3 (5)	C18—N2—Sb1—C1	−53.3 (4)
C13—C14—C15—C10	−0.7 (9)	C17—N2—Sb1—Br1	111.3 (6)
C12—C11—C16—N2	146.5 (6)	C16—N2—Sb1—Br1	−5.7 (8)
C10—C11—C16—N2	−34.9 (7)	C18—N2—Sb1—Br1	−127.9 (5)

### Hydrogen-bond geometry (Å, °)

**Table:**

*D*—H···*A*	*D*—H	H···*A*	*D*···*A*	*D*—H···*A*
N1—H1···Cl2^i^	0.86 (6)	2.39	3.220 (7)	164
C12—H12···Cl1^ii^	0.93	2.91	3.827 (6)	167
C14—H14···Cl1^iii^	0.93	2.86	3.766 (8)	164

Symmetry codes: (i) *x*−1, *y*, *z*; (ii) −*x*+1, *y*+1/2, −*z*+1/2; (iii) −*x*+1, −*y*+1, −*z*.
